# Field trial investigating the efficacy of a long-acting imidacloprid 10%/flumethrin 4.5% polymer matrix collar (Seresto®, Elanco) compared to monthly topical fipronil for the chemoprevention of canine tick-borne pathogens in Cambodia

**DOI:** 10.1016/j.crpvbd.2022.100095

**Published:** 2022-06-28

**Authors:** Lucas G. Huggins, Mark Stevenson, Zahida Baydoun, Ron Mab, Yulia Khouri, Bettina Schunack, Rebecca J. Traub

**Affiliations:** a Faculty of Veterinary and Agricultural Sciences, University of Melbourne, Parkville, Victoria, 3050, Australia; b Animal Mama Veterinary Hospital, Phnom Penh, 12312, Cambodia; c Elanco GmbH, Heinz-Lohmann-Str. 4, 27472, Cuxhaven, Germany

**Keywords:** Chemoprevention, Tick-borne pathogens, Topical ectoparasiticide, Canine health, Seresto, Fipronil

## Abstract

The tropical brown dog tick, *Rhipicephalus linnaei*, commonly infests canines in the tropics and is an important vector for disease-causing and sometimes lethal pathogens including *Babesia* spp., *Ehrlichia canis*, *Hepatozoon canis* and *Anaplasma platys*. In tropical climates ticks and their pathogens exert an extremely high infection pressure on unprotected dogs. To protect canines in such regions, effective acaricidal products possessing a speed of kill that blocks pathogen transmission is paramount. We conducted a 12-month community trial to compare the chemoprophylactic efficacy of two topical commercial acaricidal formulations: an imidacloprid 10% and flumethrin 4.5%, 8-month acting collar (Seresto®) against a monthly spot-on containing 12% w/v fipronil (Detick, Thailand). In a separate analysis, we used baseline data collected at the start of the trial to quantify tick-borne pathogen (TBP) infection status in dogs with a prior history of being administered a systemically-acting (isoxazoline) *versus* a topically-acting ectoparasiticide. We found that both topical products in the community trial demonstrated high efficacy at protecting dogs from ticks and TBP, with Seresto® demonstrating a moderate increase in protection at 3 (95% confidence interval (CI): 1–5) TBP-positive dogs per 100 dog-years at risk compared to 11 (95% CI: 4–26) TBP-positive dogs per 100 dog-years at risk for those on fipronil. Additionally, at baseline dogs treated with commercial systemic isoxazoline acaricides prior to the trial’s commencement were 2.7 (95% CI: 0.5–15.0) times more likely to be TBP-positive compared to dogs that had been topically treated, highlighting such isoxazoline products as being less efficacious than topical products at preventing canine TBP acquisition in a tropical setting.

## Highlights

• Chemopreventive products against ectoparasite infestation are essential for dog health in the tropics.
• Prevalence of canine tick-borne pathogens was higher in dogs on systemic ectoparasiticides than those on topical ones.
• Seresto® demonstrated better protection against canine tick-borne pathogen infection than a fipronil formulation.

## Graphical abstract

**Figure:**
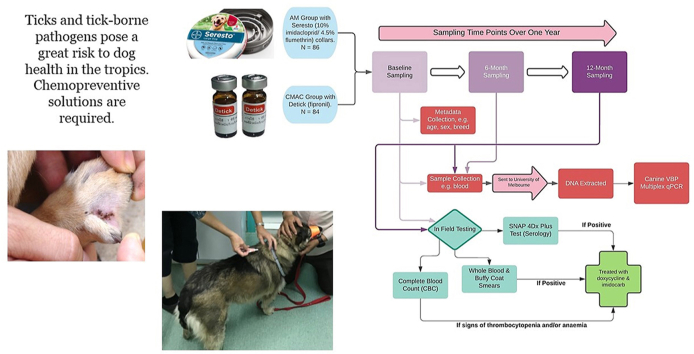

## 1 Introduction

Ticks are important blood-feeding arthropods that represent a large threat to dog populations worldwide due to their capacity to vector a range of viruses, bacteria, and protozoa, collectively known as tick-borne pathogens (TBP) (Chomel, 2011; Brianti et al., 2013; Fourie et al., 2013a; Colella et al., 2020). The impacts of TBP are particularly pernicious, given tick species such as the brown dog tick, *Rhipicephalus sanguineus* (*sensu lato*), are monotropic three-host ticks, meaning that local ectoparasite numbers can rapidly increase and facilitate the easy spread of TBP (Dantas-Torres, 2010; Gray et al., 2013). Ticks including *R. sanguineus* (*s.l.*) and, in the tropics, *Rhipicephalus linnaei*, transmit pathogens that can be lethal to dogs, for example *Ehrlichia canis*, the aetiological agent of canine monocytic ehrlichiosis (CME). At the same time, diseases caused by protozoans such as *Babesia vogeli* and *Hepatozoon canis* can also inflict significant morbidity (Little, 2010; Chomel, 2011; Koh et al., 2016; Šlapeta et al., 2021).

In the tropics, relatively high levels of humidity and mean annual temperatures facilitate the spread of TBP as local vectors do not experience strong population fluctuations that occur in parts of the world with distinct seasonal cycles (Galay et al., 2018; Snellgrove et al., 2020). Additionally, in low-to-middle income countries, where large numbers of semi-domesticated or free-roaming dogs have limited access to veterinary care, conditions are often further conducive for TBP propagation (Inpankaew et al., 2016; Koh et al., 2016; Colella et al., 2020).

Given ectoparasites and the TBP they transmit can generate severe health impacts in dogs, effective chemopreventive medications and products that block TBP transmission are critically important. Topical products that act from the animal’s hair and dermis to repel ectoparasites and rapidly kill them are particularly beneficial as they prevent vector haematophagy and thus do not provide a time period over which TBP can be transmitted (Dantas-Torres et al., 2013; Schorderet-Weber et al., 2017). Such benefits are further highlighted when their effectiveness is compared to ectoparasiticides which rely on systemic uptake of the active ingredient into the host’s tissues, e.g. isoxazoline compounds. Systemic ectoparasiticides are ingested at the point of vector feeding, subsequently killing the ectoparasite whilst at the same time also providing a timeframe over which some TBP can be transmitted (Ohmes et al., 2015; Jongejan et al., 2016). The TBP blocking ability of topical products that rely on chemicals present on the skin of the dog, thereby repelling and/or quickly killing ectoparasites, has been experimentally demonstrated *via* challenge infections to be superior to products that rely on systemic chemical uptake for the prevention of TBP transmission, e.g. for *E. canis* (Jongejan et al., 2016; Schorderet-Weber et al., 2017).

An imidacloprid 10% and flumethrin 4.5% vinyl collar (Seresto®, Bayer) designed to provide long-term protection from ticks and fleas for up to 8 months has been tested for its ability to repel and kill ectoparasites as well as to prevent the transmission of some TBP (Stanneck et al., 2012c; Krüdewagen et al., 2015). The two active chemicals in Seresto® collars act synergistically to augment the neurotoxic effects of either alone, on insect and arachnid nervous systems (Stanneck et al., 2012a). A further advantage of these collars is their purported 8-month efficacy, which results in improved owner compliance when compared to products which offer shorter-term protection and therefore need regular administration (Stanneck et al., 2012c; Dantas-Torres et al., 2013; Ohmes et al., 2015).

Seresto® collars have been shown to prevent flea and tick infestations with high efficacy (90–100%) for up to 8 months, even in regions with high ectoparasite pressure or within canine populations with regular exposure to rain and other environmental factors that might negatively impact on product effectiveness (Stanneck et al., 2012b, 2012c; Brianti et al., 2013; Dantas-Torres et al., 2013; Halos et al., 2014; Beugnet et al., 2018). Moreover, Seresto® collars have been shown to be efficacious at preventing the transmission of key canine TBP, including *E. canis* (Stanneck & Fourie, 2013), *Babesia canis* (Fourie et al., 2013b), *Borrelia burgdorferi* (*sensu lato*) and *Anaplasma phagocytophilum* (Krämer et al., 2020) with the last two being important zoonotic agents for which canines act as reservoirs.

Less contemporary topical ectoparasiticides, such as fipronil, a phenyl pyrazole that has been used on canines since the early 1990s have also been extensively tested to assess their ability to prevent infestation and block TBP transmission. Prior field studies have found fipronil in commercial topical formulations such as Frontline™ (Boehringer Ingelheim) to demonstrate efficacies as high as 94–97% for preventing flea infestations and 94–100% for stopping tick infestations, concluding that it is a highly efficacious topical ectoparasiticide (Kužner et al., 2013; Rohdich et al., 2014). Moreover, such infestation prevention also translates into an ability to block TBP contraction, with fipronil having been shown to prevent transmission of *E. canis* by *R. sanguineus* (*s.l.*) in areas of endemicity and high pathogen pressure in Djibouti (Davoust et al., 2003).

Field efficacy studies have also been conducted on Seresto®, predominantly in Europe, with research focusing on testing efficacy at repelling the relevant vectors and blocking the associated TBP found in these regions (Stanneck et al., 2012c; Brianti et al., 2013, 2014; Dantas-Torres et al., 2013). Nonetheless, in tropical parts of the Asia-Pacific pressure from ectoparasites and the prevalence of TBP in local dog populations can greatly exceed those found in temperate regions. For example, prior studies in Italy have shown Seresto® collars to be efficacious at blocking *B. vogeli* transmission with an estimated local prevalence of 5–6% for this pathogen (Cassini et al., 2009; Dantas-Torres et al., 2013). In contrast, *B. vogeli* has been found in as many as 33% of free-roaming dogs in Cambodia (Inpankaew et al., 2016), 23% of dogs in Cambodia’s capital, Phnom Penh, and 9–13% of dogs in Thailand (Liu et al., 2016; Huggins et al., 2019, 2021a) and 44% of dogs in remote communities throughout parts of tropical and arid regions of Australia (Barker et al., 2012). Such high pathogen pressure environments provide an ideal setting within which to test Seresto® collars to elucidate whether a high level of ectoparasite repellence and TBP-blocking efficacy is still maintained. Comparing Seresto®’s efficacy to fipronil, a topical ectoparasiticide that has been demonstrated as effective at preventing tick infestation and is readily available throughout Southeast Asia, provided a benchmark from which to assess the potential TBP-blocking efficacy Seresto® confers.

With this background, we report the methodology and findings from a two-part study designed to document the risks of TBP infection in dogs with a history of using either topical or systemic ectoparasiticides and, investigate the efficacy of two topical ectoparasiticides on blocking canine TBP transmission in Cambodia. First, we conducted a baseline assessment of dogs to determine the prevalence of TBP infection in those being treated with topical and systemic ectoparasiticides as well as those with no prior ectoparasiticide treatment. Secondly, we carried out a 12-month prospective superiority community trial on dogs enrolled into the baseline study to compare the incidence of newly acquired TBP in dogs treated with two different topical commercial ectoparasiticides: Seresto® collars and fipronil (12% w/v) (Detick, Thailand), the latter being a topical ectoparasiticide commonly used in Southeast Asia.

## 2 Methods

### 2.1 Study design and study area

We conducted a community trial where our primary aim was to compare the chemoprophylactic efficacy of two topical commercial acaricidal formulations: an imidacloprid 10% and flumethrin 4.5%, 8-month acting collar (Seresto®) against a monthly spot-on containing 12% w/v fipronil (Detick, Thailand), administered according to labelled instructions.

Two communities of dogs were selected for investigation in Cambodia between January and March 2020. The first comprised dogs (pets) that were owned by clients of the Animal Mama Veterinary Hospital in Phnom Penh (11°31′N, 104°55′E) and dogs from the Home of Heroes animal shelter in Siem Reap (13°26′N, 103°45′E). The second community comprised dogs that worked for and were cared for by the Cambodian Mine Action Centre (CMAC). This group included dogs that were kenneled and used for breeding as well as individuals regularly taken into the field to assist with landmine decontamination programmes at three locations across the country: Phnom Penh city (11°31′N, 104°55′E), an urban environment; Kampong Chhnang (12°15′N, 104°39′E), a rural town; and Battambang (13°25′N, 103°73′E), a medium-sized city. Dogs in the first community, owned by clients of Animal Mama Veterinary Hospital, were recruited by e-mail or over the phone in January 2020. A written description of the study together with contact details of investigators and expectations of each dog owner were provided in English and/or Khmer along with a consent form for signing. Official permission was obtained by the Director General of CMAC in January 2019 to enroll dogs from the second community into this study.

Animals were deemed eligible for enrolment if they were over 8 weeks of age on March 1, 2020, if they were walked outdoors a minimum of three times per week, were clinically normal on physical examination and were expected to continue living in Cambodia for the next 12 months. Dogs that were previously administered ectoparasiticide products were only enrolled into the study after the window of labelled efficacy for the applied or administrated product had ceased. Enrolled animals were tested for a pre-existing TBP infection *via* serology, blood smear examination and qPCR and allowed to complete a treatment course before starting the community trial (proceeding sections include details of diagnostics used and treatments initiated).

The trial was conducted between January 2020 and July 2021 for dogs recruited through Animal Mama Veterinary Hospital and from March 2020 to March 2021 for the CMAC dogs. Dogs in the first community (clients of the Animal Mama Veterinary Hospital in Phnom Penh and clients from the Home of Heroes animal shelter in Siem Reap) received Seresto® collars (Bayer) and are referred to as the Investigational Veterinary Product (IVP) group hereon in. Dogs in the second community (dogs that worked for and were cared for by the CMAC) received monthly treatment with fipronil and are referred to as the Control Product (CP) group. Both of these products act topically with the active compound working from the subject’s skin or hair.

On the prospective community trial’s date of commencement, a Seresto® collar was fitted to each of the dogs in the IVP group. Dogs in the CP group received the first of their monthly fipronil treatment topically between the shoulder blades as per the manufacturer’s instructions. For the IVP group the Seresto® collars were replaced after 8 months, or earlier if the collar detached and was lost (within a 24-h period).

A community trial study design was necessary because ectoparasiticide treatment could not be randomly allocated to the pool of eligible dogs. This was because over this study period, the CMAC dogs had to continue their regular demining activities and had a strict ectoparasite and endoparasite prevention and treatment protocol that was not permitted to be altered by the organisation for the sake of this study.

**Fig. 1:**
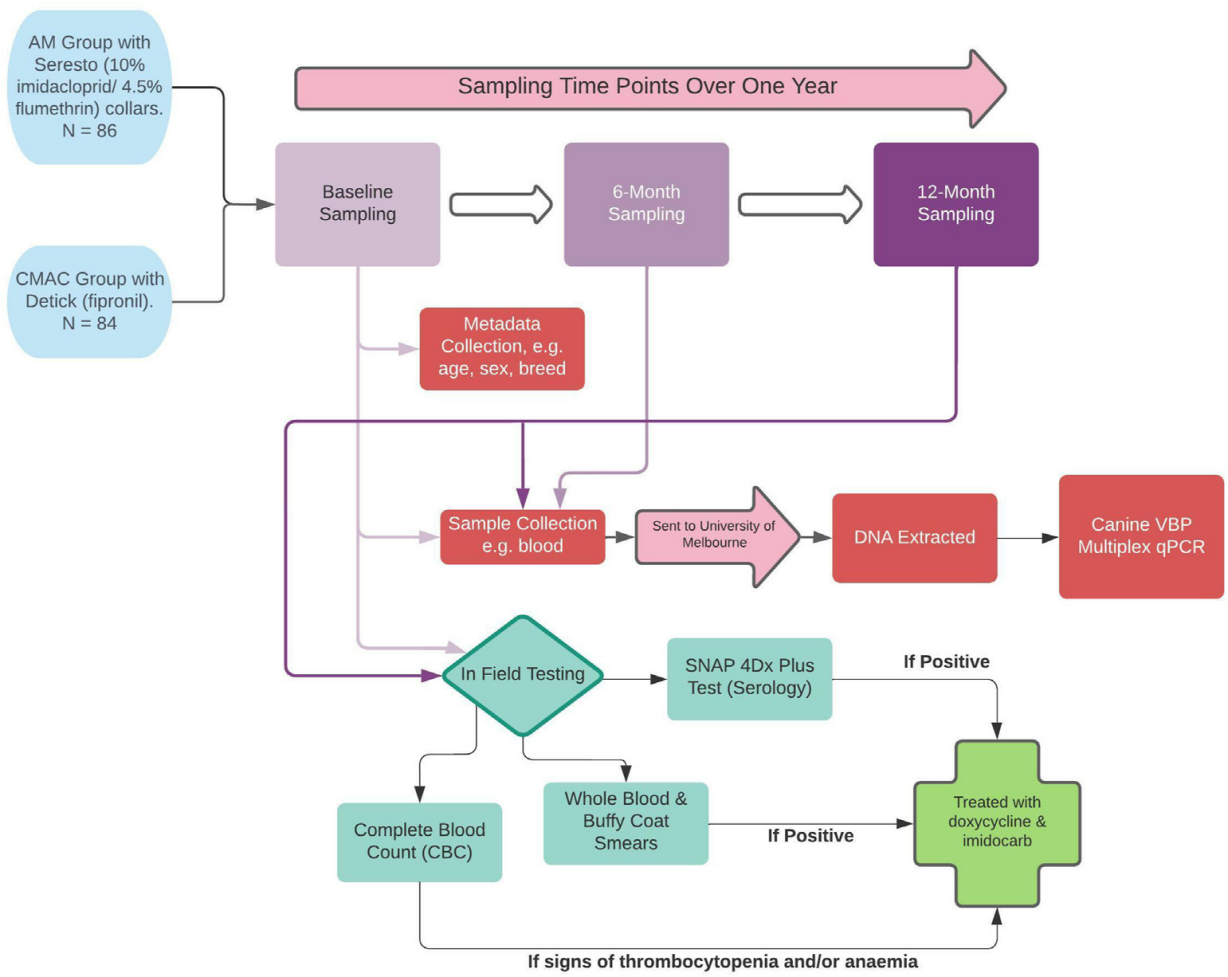
Experimental design for (i) baseline assessment where metadata collected were used to compare rate of tick-borne pathogen (TBP) positivity in canines treated with systemic, topical and no ectoparasiticides prior to the study’s commencement and (ii) prospective community trial comparing two topical ectoparasiticides (Seresto® and Detick) for prevention of TBP contraction.

Dogs enrolled into the prospective community trial were followed by investigators for a period of 12 months. A 6-month and 12-month follow-up period was used to assess treatment efficacy. Fig. 1 provides a diagrammatic representation of the sequence of treatment and testing events carried out as part of the baseline assessment and prospective community trial.

Throughout the follow-up period dogs in the CP group (i.e. CMAC dogs) were kept at centres comprised of large areas of open land, fields and trees. They had access to these sites during the day for training activities. During evenings and nights these dogs were kept in raised cages off the ground. The CMAC centres and grounds were not closed environments and wildlife and occasionally community dogs were able to enter such areas resulting in opportunity for ectoparasite exposure. Dogs in the IVP group were continued to be cared for by their respective owners who were encouraged to continue walking their dogs outside of their premises in areas frequented by community dogs, as they were prior to the study’s commencement.

### 2.2 Sampling and diagnostic methods

At enrolment, dogs were subject to a complete physical examination by the study’s veterinarians. Each animal’s age, sex, breed, neutering status, whether imported, prior ectoparasite and endoparasite control history as well as other ongoing medication details were recorded. Previous ectoparasite and endoparasite control product used with particular focus on whether a topical, systemic or no ectoparasiticide had been administered prior to the study’s commencement was key information collected at this baseline assessment point. Furthermore, ectoparasiticide product compliancy was noted, with compliancy defined as either poor, intermittent or high depending on the adherence to manufacturer recommendations. Compliancy was defined as poor if the owner only applied parasiticides when taking their dog to occasional veterinary check-ups, compliancy was intermittent if a product was used but with gaps over the product’s labelled window of efficacy, compliancy was high if the parasiticide was used as per the product’s labelled instructions.

Dogs were then subjected to a detailed physical exam by the study veterinarians. Two millilitres of samples were drawn *via* cephalic or jugular venipuncture from each dog into two EDTA tubes. Blood was subjected to a complete blood count (CBC), examination for TBP using stained whole and buffy coat smears and SNAP 4Dx Plus (IDEXX, Westbrook, ME, USA) to test for the presence of antibodies to *Anaplasma* spp., *E. canis*, *B. burgdorferi*, and antigens of *Dirofilaria immitis*. The second tube of whole blood was frozen at −20 °C and transported on ice to the Melbourne Veterinary School at the University of Melbourne. At the Melbourne Veterinary School, DNA extraction was conducted using a DNeasy Blood & Tissue Kits (Qiagen, Hilden, Germany) and subject to a previously developed multiplex real-time (qPCR) assay for common canine vector-borne pathogens for the region (Huggins et al., 2021b). Data were collected on the positivity status for the pathogens *Anaplasma platys*, *Babesia gibsoni*, *B. vogeli*, *E. canis* and *H. canis*. Collection of blood for qPCR analysis was also conducted at the 6- and 12-month examination time-points.

### 2.3 Clinical and safety observations

Throughout the follow-up period dogs were examined at monthly intervals for clinical signs consistent with TBP infection as well as potential adverse effects to their chemoprophylaxis treatment (e.g. dermatological changes at the site of application). Dogs were also checked for the presence of ticks at common feeding sites, e.g. head, ears, inter-digital regions, with those found collected and preserved in 80% ethanol. At this time, the integrity of the Seresto® collars were also confirmed and CP dogs administered fipronil. Owners were also questioned to ensure compliance with the study protocol as per eligibility criteria at enrolment.

### 2.4 Treatment and rescue treatment

Dogs that were found to have subclinical anaemia or thrombocytopenia at the time of enrolment, and/or those found exposed to a TBP *via* SNAP 4Dx Plus testing or positive *via* blood smears were put on a course of doxycycline 10 mg/kg SID. The course of doxycycline was for 14 days if positive to *A. platys*, whilst a 28-day course was used for *E. canis*. At baseline, dogs found to be anaemic and/or thrombocytopenic were administered imidocarb dipropionate 5–7 mg/kg IM over a 2-week interval to clear any potential concurrent *B. vogeli* infection. Dogs were only progressed forward into the prospective community trial after their treatment course had been completed.

Rescue treatment was also initiated using the treatment protocols described for enrolled dogs found to be vector-borne pathogen positive by smear or qPCR at any of the follow-up examinations (6 and 12 months). Additionally, dogs found positive to an apicomplexan were treated with imidocarb dipropionate 5–7 mg/kg IM twice, 2 weeks apart to ensure TBP negativity (Sainz et al., 2015).

### 2.5 Data handling and statistical analysis

Sample size calculations conducted for the prospective community trial were carried out to determine the number of dogs that needed to be enrolled into the study to provide sufficient power to demonstrate that the confidence limits for the difference between the two treatments excluded zero. This takes the assumption that the TBP prevalence for the IVP group was less than the TBP prevalence for the CP group by an amount called the smallest clinically important difference, delta. Given 80% power, a 95% confidence level at 5% of significance, an estimated TBP prevalence of 10% and 30% in the IVP and CP group, respectively, and a smallest clinically important difference between treatment groups of 5%, the total sample size of dogs needing to be enrolled and complete the study was 166. In addition to this, a study drop-out of 15% was estimated based on prior data and conversations with CMAC and Animal Mama regarding regular adoption of CMAC canines or movement of clients and dogs to other regions of the country, as well as dog mortality. Hence, minimum sample size required was increased to 195 dogs. Sample size calculations were carried out using the ‘sample size for a cohort study’ function in EpiTools (https://epitools.ausvet.com.au). The sample size required for the baseline assessment was calculated using the ‘Sample size to detect a significant difference between two proportions’ function in EpiTools and was found to be less than that required for the prospective community trial hence the community trial’s sample size was assessed to be suitable for both parts of this investigation (Sergeant, 2018).

The prevalence of TBP infection, as determined by qPCR results and TBP exposure as determined by serology, was calculated using the epi.directadj function in the contributed *epiR* package (Breslow & Day, 1987; Stevenson et al., 2021) in R (R Development Core Team, 2021). These calculations were used to compare dogs on systemic, topical or no ectoparasiticide products prior to the study’s commencement, directly adjusting for the confounding effect of the environment in which dogs were kept (urban, rural). This provided TBP prevalence estimates that were comparable, given that the environment in which a dog is kept can give rise to markedly different TBP infection pressures (Gracia et al., 2008; Farkas et al., 2009; Smith et al., 2011). Dogs that were on a treatment that had been administered with poor compliance, see Section 2.2*.* ‘Sampling and diagnostic methods’, were included in the no ectoparasiticide category for this analysis, whilst those administered their ectoparasiticide with moderate or high compliance were included in their respective product group. Baseline assessment TBP crude (i.e. unadjusted) prevalence estimates for systemic, topical and no ectoparasiticide-treated dogs and their 95% confidence intervals (CI) were presented as error barplots, using the data from both the qPCR and serological analyses. In addition, using the qPCR data, calculations were carried out to identify the number of dogs needed to be treated for benefit (NNTB), defined as the inverse of the attributable risk (the directly adjusted probability of a dog on a topical ectoparasiticide being TBP-positive minus the directly adjusted probability of a dog on a systemic ectoparasiticide being TBP-positive). NNTB estimates were interpreted as the number of dogs that would need to be treated with a topical ectoparasiticide to prevent one additional dog from being TBP-positive. 95% CI for the NNTB estimates are reported using the approach described by (Altman, 1998).

For the prospective community trial comparing the topical ectoparasiticides Seresto® and Detick for the 12-months’ qPCR data, the frequency of TBP positivity for the CP and IVP groups were expressed as incidence rate measures, that is, the number of incident TBP cases divided by the total number of dog-trial days at risk. This approach allowed for the possibility of owners withdrawing their dogs from the study or for dog dropout due to death or sickness prior to the study completion date. Again, the epi.directadj in *epiR* was used, adjusting for the confounding effect of the environment in which a dogs was kept. Crude TBP incidence rate estimates for IVP (Seresto®)- and CP (Detick)-treated dogs and their 95% CI were presented as error barplots. NNTB calculations were also conducted and interpreted as the number of dogs that would need to be treated with the IVP ectoparasiticide (Seresto®) to prevent one additional dog from becoming TBP-positive.

Missing data caused by dog dropout due to sickness, death, or owner removal from the trial for reasons unconnected with the trial itself were managed statistically, so as to avoid biasing our model estimates of treatment effect (Shih, 2002; Jakobsen et al., 2017; McNeish, 2017). We used multiple imputation with chained equations (MICE) to impute the likely TBP qPCR positivity status of dogs that dropped out of the study before they were tested at either 6 or 12 months using the contributed *MICE* package in R. Analyses were conducted on the imputed dataset using the approach described above.

Additionally, for the prospective community trial the IVP (Seresto®) collar efficacy at preventing TBP infection ($ETBP_{IVP}$) was calculated as follows:

Equation 1 $$ETBP_{IVP}=\;\left(\frac{P_{CP}-\;P_{IVP}\;}{P_{CP}}\right)\;\times 100$$

In Equation 1, $P_{CP}$ and $P_{IVP}$ equal the proportion of dogs in the CP group and IVP group that were newly TBP pathogen-positive at the end of the follow-up period, respectively.

The efficacy of the IVP (Seresto®) collar against ectoparasite infestation ($EEP_{IVP}$) was calculated using a similar approach:

Equation 2 $$EEP_{IVP}=\;\left(\frac{EP_{CP}-\;EP_{IVP}\;}{EP_{CP}}\right)\;\times 100$$

where $EP_{CP}$and $EP_{IVP}$ equal the mean number of ectoparasites found attached to dogs in the CP group and IVP group at the end of the follow-up period, respectively. The IVP group’s Seresto® collars were deemed effective against TBP and ectoparasites if the calculated efficacy, based on means, was at least 90% (Marchiondo et al., 2013). The Chi-square test was used to assess whether there was a statistically significant difference in the number of dogs that dropped out of the study between the CP and IVP groups within the prospective community trial.

## 3 Results

### 3.1 Baseline assessment comparing topical and systemic ectoparasiticides

A total of 186 dogs were comparable within the baseline assessment that had information provided on ectoparasiticide usage prior to the study’s commencement (*n* = 118 on topical treatments and *n* = 61 on systemic treatments, *n* = 7 on no ectoparasiticide).

Key information collected at baseline apart from the type of ectoparasiticide used prior to the study’s commencement was the compliancy of product usage, as defined in Section 2. No differences were observed in moderate or high owner compliancy categories between dogs being treated with systemic or topical ectoparasiticides, whilst those on poorly compliant regimes were included in the no ectoparasiticide used group for subsequent analysis. The majority of dogs on a systemic chemopreventive were using an isoxazoline product, such as Bravecto® (MSD Animal Health) that uses the active ingredient fluralaner, and Nexgard® (Boehringer Ingelheim) which uses the active ingredient afoxolaner.

At baseline, two dogs on systemic ectoparasiticides were identified as ectoparasite-positive whilst one individual on a topical ectoparasiticide was ectoparasite-positive (a tick). Of the seven dogs that were not on an ectoparasiticide, five were found infested with ectoparasites of which all had ticks and one also had fleas. Using the canine TBP-targeting multiplex qPCR we found that five dogs on systemic products were positive for a TBP, whilst for dogs on topical products two were positive. Of the dogs on no ectoparasiticides, two were TBP-positive (see Table 1 for details of the TBP species identified). All TBP-positive individuals in both groups were identified using qPCR with no pathogens detected *via* whole blood or buffy coat smears.

**Table 1:** Between group comparison of the number of qPCR positive dogs (+) and apparent pathogen prevalence (%) with 95% confidence interval (CI) found for single, mixed and overall infections at study baseline

Pathogen	Systemic products (*n* = 61)		Topical products (*n* = 118)		No ectoparasiticide (*n* = 7)	
	+	% (95% CI)	+	% (95% CI)	+	% (95% CI)
Single and mixed infection						
*Anaplasma platys*	1	2 (0–9)	2	2 (0–6)	2	29 (8–64)
*Babesia vogeli*	2	3 (0–11)	0	0 (0–3)	1	14 (3–51)
*Ehrlichia canis*	1	2 (0–9)	1	1 (0–5)	0	0 (0–35)
*Hepatozoon canis*	1	2 (0–9)	0	0 (0–3)	0	0 (0–35)
Mixed infection						
*A. platys* + *E. canis*	0	0 (0–6)	1	1 (0–5)	0	0 (0–35)
*A. platys* + *B. vogeli*	0	0 (0–6)	0	0 (0–3)	1	14 (3–51)
Total positive	5	8 (4–18)	2	2 (0–6)	2	29 (8–64)
Total negative	56	92 (82–96)	116	98 (94–100)	5	71 (36–92)

*Notes*: Whether a dog had been given a systemic, topical or no ectoparasiticide product by their owner prior to baseline data collection was ascertained by a veterinarian’s collection of a detailed history for all dogs enrolled. No dogs were found *B. gibsoni*-positive.

**Fig. 2:**
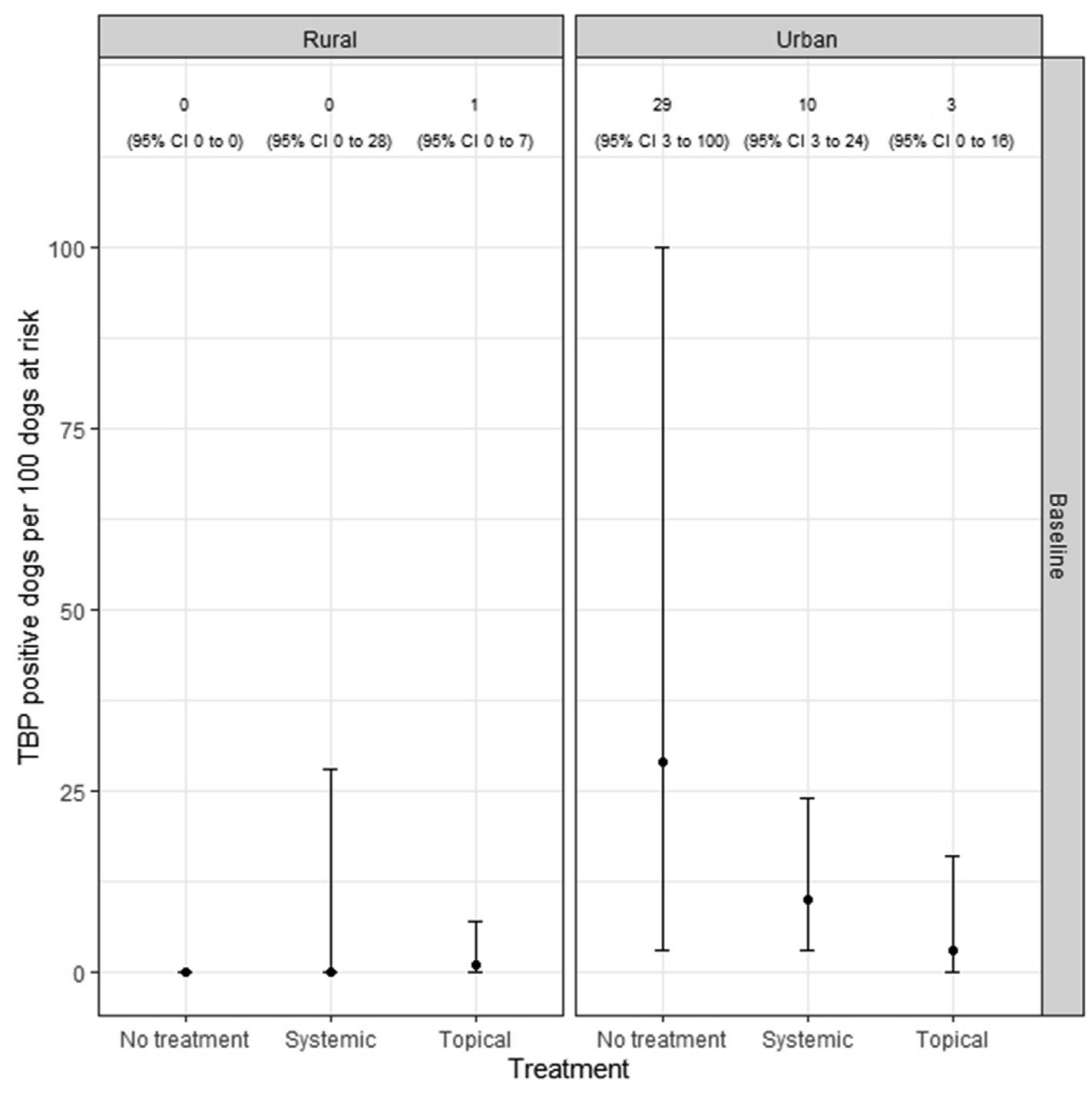
Error barplot showing crude (unadjusted) tick-borne pathogen (TBP) apparent prevalence estimates as assessed by qPCR for dogs treated with systemic, topical and no ectoparasiticide products, conditioned by environment (urban, rural). TBP prevalence is expressed as the number of TBP positive dogs per 100 dogs at risk. The error bars show the lower and upper bounds of the 95% confidence interval (CI) for the TBP prevalence estimates.

Figure 2 shows the crude apparent TBP prevalence estimates for systemic-, topically- and no ectoparasiticide-treated dogs, conditioned by the environment in which the dog was kept (urban, rural). After adjusting for the effect of environment, there were 5 (95% CI: 2–20) TBP-positive systemically treated dogs per 100 dogs at risk, compared with 2 (95% CI: 0–9) TBP-positive topically treated dogs per 100 dogs at risk. This result can be alternatively expressed as an apparent prevalence risk ratio, i.e. dogs that were systemically treated were 2.7 (95% CI: 0.5–15) times more likely to be TBP-positive compared with dogs that were topically treated. For dogs on no ectoparasiticide product there were 14 (95% CI: 2–52) TBP-positive dogs per 100 dogs at risk. After accounting for the confounding effect of environment, we estimate that 31 (95% CI: 8–66) dogs needed to be treated topically to avoid one case of TBP.

The same canine blood samples were also serologically tested which identified 22 (36%, 95% CI: 25–49%) TBP-exposed dogs on systemic products and 16 (14%, 95% CI: 9–21%) on topical products (Table 2). Dogs that were on no ectoparasiticides had higher relative levels of TBP exposure with three of seven dogs serologically-positive. Positivity was only found for the pathogens *A. platys* and *E. canis* across all the dogs tested, see Table 2 for details of the apparent prevalence of different TBP identified.

**Table 2:** Between group comparison of the number of serologically positive dogs (+) and apparent pathogen prevalence (%) with 95% confidence interval (CI) found for single, mixed and overall infections, as detected by SNAP 4Dx Plus Test at study baseline

Pathogen	Systemic products (*n* = 61)		Topical products (*n* = 118)		No ectoparasiticide (*n* = 7)	
	+	% (95% CI)	+	% (95% CI)	+	% (95% CI)
Single and mixed infection						
*Anaplasma* spp.	17	28 (18–40)	11	9 (5–16)	2	29 (8–64)
*Ehrlichia* spp.	14	22 (14–35)	8	7 (3–13)	2	29 (8–64)
Mixed infection						
*Anaplasma* + *Ehrlichia*	9	15 (8–26)	3	3 (1–7)	1	14 (3–51)
Total positive	22	36 (25–49)	16	14 (9–21)	3	43 (16–75)
Total negative	39	64 (51–75)	102	86 (79–91)	4	57 (25–84)

*Notes*: Whether a dog had been given a systemic, topical or no ectoparasiticide product by their owner prior to baseline data collection was ascertained by a veterinarian’s collection of a detailed history for all dogs enrolled. No dogs were *B. burgdorferi*- or *D. immitis*-positive.

**Fig. 3:**
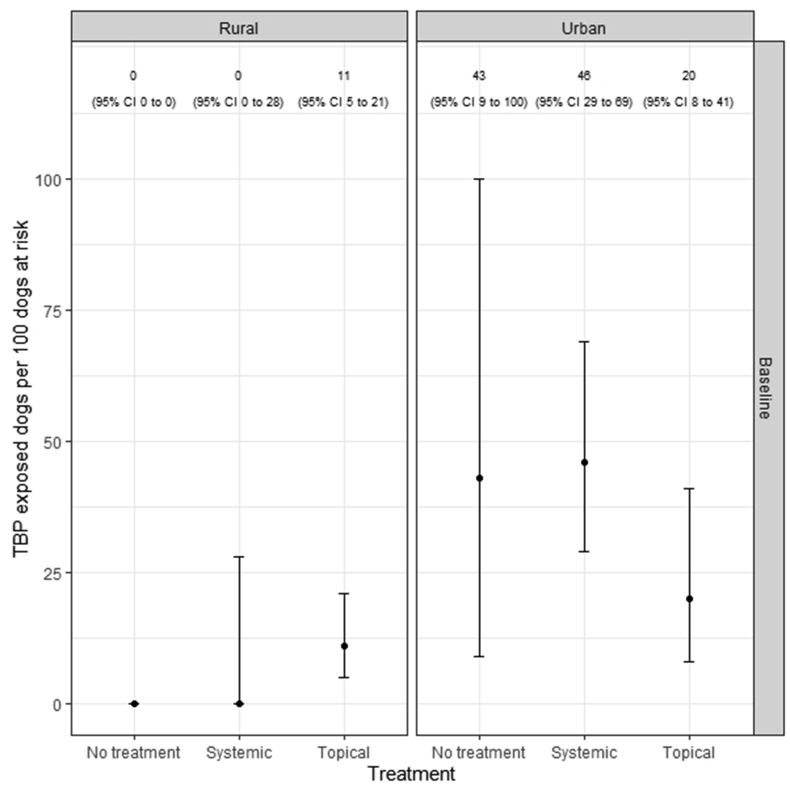
Error barplot showing crude (unadjusted) apparent vector-borne pathogen (TBP) exposure prevalence estimates as assessed by serology for dogs treated with systemic, topical and no ectoparasiticide products, conditioned by environment (urban, rural). TBP prevalence is expressed as the number of TBP exposed dogs per 100 dogs at risk. The error bars show the lower and upper bounds of the 95% confidence interval (CI) for the TBP prevalence estimates.

Figure 3shows the crude apparent TBP serological exposure prevalence estimates for systemic-, topical- and no ectoparasiticide-treated dogs, conditioned by the environment in which the dog was kept (urban, rural). After adjusting for the effect of environment, the apparent prevalence of TBP-exposure was 23 (95% CI: 14–40) per 100 dogs at risk for those systemically treated, compared with an apparent prevalence of 15 (95% CI: 8–27) per 100 dogs at risk for those topically treated. For dogs on no ectoparasiticide product, the apparent prevalence of TBP exposure was 21 (95% CI: 4–63) per 100 dogs at risk.

### 3.2 Prospective community trial comparing two topical ectoparasiticides

A total of 197 dogs met the eligibility criteria and were enrolled into the prospective community trial which was greater than the minimum required (*n* = 166) to accommodate drop-out of study subjects. The number of dogs that completed the prospective community trial to the study end was 164 (*n* = 83 in the CP group and *n* = 81 in the IVP group), i.e. 33 dogs dropped out due to sickness unrelated to the trial, mortality unrelated to the trial or clients having left the study locations with their animals. There was no statistically significant difference in the number of dogs that dropped out of the community trial in the IVP and CP groups at the *P* < 0.05 level as determined by a Chi-square test (*χ*^2^ = 0.1005, *n* = 197, *df* = 1, *P* = 0.751).

The composition of dogs enrolled into the prospective community trial by age and sex was similar for both treatment groups (Table 3) while there were more purebred dogs in the CP group compared with the IVP group due to CMAC favouring the use of Belgian Malinois to conduct landmine decontamination. In the IVP group dog breeds were more varied including Beagles, Bichon Frise, Chihuahuas, Cocker Spaniels, Dachshunds, German Shepherds, Golden Retrievers Labradors, and Poodles. There were greater numbers of neutered dogs in the IVP group compared with the CP group (Table 3). Dogs in the IVP group were from Phnom Penh (PP, 81%) and Siem Reap (SR, 19%) while dogs in the CP group were from Kampong Chhnang (KC, 80%), Battambang (BB, 15%) and PP (5%).

**Table 3:** Relative composition of canine sex, age, breed and neutering status in the IVP (Seresto®) group and CP (Detick) group

Sex	IVP Group	CP Group	Total	Neutered	IVP Group	CP Group	Total
Female	44 (54%)	35 (42%)	79	Yes	30 (37%)	0 (0%)	30
Male	37 (46%)	45 (54%)	82	No	19 (23%)	71 (86%)	90
Unreported	0 (0%)	3 (4%)	3	Unreported	32 (40%)	12 (14%)	44
Total	81	83	164	Total	81	83	164
Age	IVP Group	CP Group	Total	Breed	IVP Group	CP Group	Total
< 6 months	6 (7%)	0 (0%)	6	Local	35 (43%)	0 (0%)	35
> 6 months < 12 months	16 (20%)	13 (16%)	29	Cross	13 (16%)	14 (17%)	27
> 12 months	52 (64%)	69 (83%)	121	Pure	33 (41%)	67 (81%)	100
Unreported	7 (9%)	1 (1%)	8	Unreported	0 (0%)	2 (2%)	2
Total	81	83	164	Total	81	83	164

*Note*: Local dog breeds refer to mongrels that do not have obvious recognisable characteristics of a common dog breed.

At the 6-month time-point two dogs in the IVP group were found with ticks on them (2%; 95% CI: 0.6–8.0%), whilst one dog in the CP group was found with a tick (1%; 95% CI: 0.2–6.0%), see Table 4. At the 12-month time-point, two dogs in the CP cohort were found with ticks (2%; 95% CI: 0.7–8.0%) but no ectoparasites were found in the IVP cohort (Table 4). Nonetheless, at all time-points, none of the ticks found were attached or engorged.

**Table 4:** Between group comparison of the number of dogs found positive to TBP *via* qPCR with 95% confidence interval (CI) at the 6-month and 12-month time-points in the prospective community trial

	Seresto (*n* = 81)	Fipronil (*n* = 83)	Total (*n* = 164)
Ticks found on dogs			
6-months	2 (0.6–8.5)	1 (0–6.5)	3 (0.6–5.2)
12-months	0 (0–5.0)	2 (0.6–8.3)	2 (0.3–4.3)
Total	2 (0.6–8.5)	3 (1.2–10.0)	5 (1.3–6.9)
% Infested	2.5 (1.0–9.0)	3.6 (1.0–10.0)	3.0 (1.0–7.0)
% Negative	97.5 (91.0–99.0)	96.4 (90.0–99.0)	97 (93.0–99.0)
TBP-infected dogs			
6-months	1 (0.2–6.7)	2 (0.6–8.3)	3 (0.6–5.2)
12-months	3 (1.2–10.0)	4 (1.9–11.7)	7 (2.1–8.6)
Total	4 (1.9–12.0)	6 (3.4–14.9)	10 (3.4–10.9)
% Infected	4.9 (2.0–12.0)	7.2 (3.0–15.0)	6.1 (3.0–11.0)
% Negative	95.1 (88.0–98.0)	92.8 (85.0–97.0)	93.9 (89.0–97.0)

*Note*: The only ectoparasites found on dogs at any time point were ticks and these were never observed as attached or engorged.

At the 6-month follow-up one IVP group dog was found to have acquired a new TBP infection with *A. platys* by qPCR (1%, 95% CI: 0.2–6.0%), whilst two CP dogs were found newly TBP-positive (2%, 95% CI: 0.6–8.0%), of these one was positive to *A. platys* and one to *E. canis* (both 1%, 95% CI: 0.2–6.0%), see Table 4. All dogs initially found TBP-positive or anaemic at baseline that were treated for a bacterial TBP infection were found TBP-negative at 6-months, indicating successful treatment with doxycycline.

At the 12-month follow-up, three dogs were found to have acquired new TBP infections in the IVP group, comprising one new infection with *B. vogeli*, one with *H. canis* and one with *A. platys* (all 1%, 95% CI: 0.2–7.0%). In comparison in the CP group, four dogs had contracted a new TBP, two with *B. vogeli* and two with *A. platys* (both 2%, 95% CI: 0.7–8.0%), see Table 4. TBP blocking efficacy of the IVP, as defined by the equation in Section 2.5, was 49% between 0 and 6 months and 23% between 6 and 12 months and therefore was not deemed substantially more effective at blocking TBP than the fipronil used in the CP group as defined by Marchiondo et al. (2013). In contrast, the ectoparasite prevention ability was 100% for the IVP, given that no ticks were found attached or engorged in either group at any time-point.

**Fig. 4:**
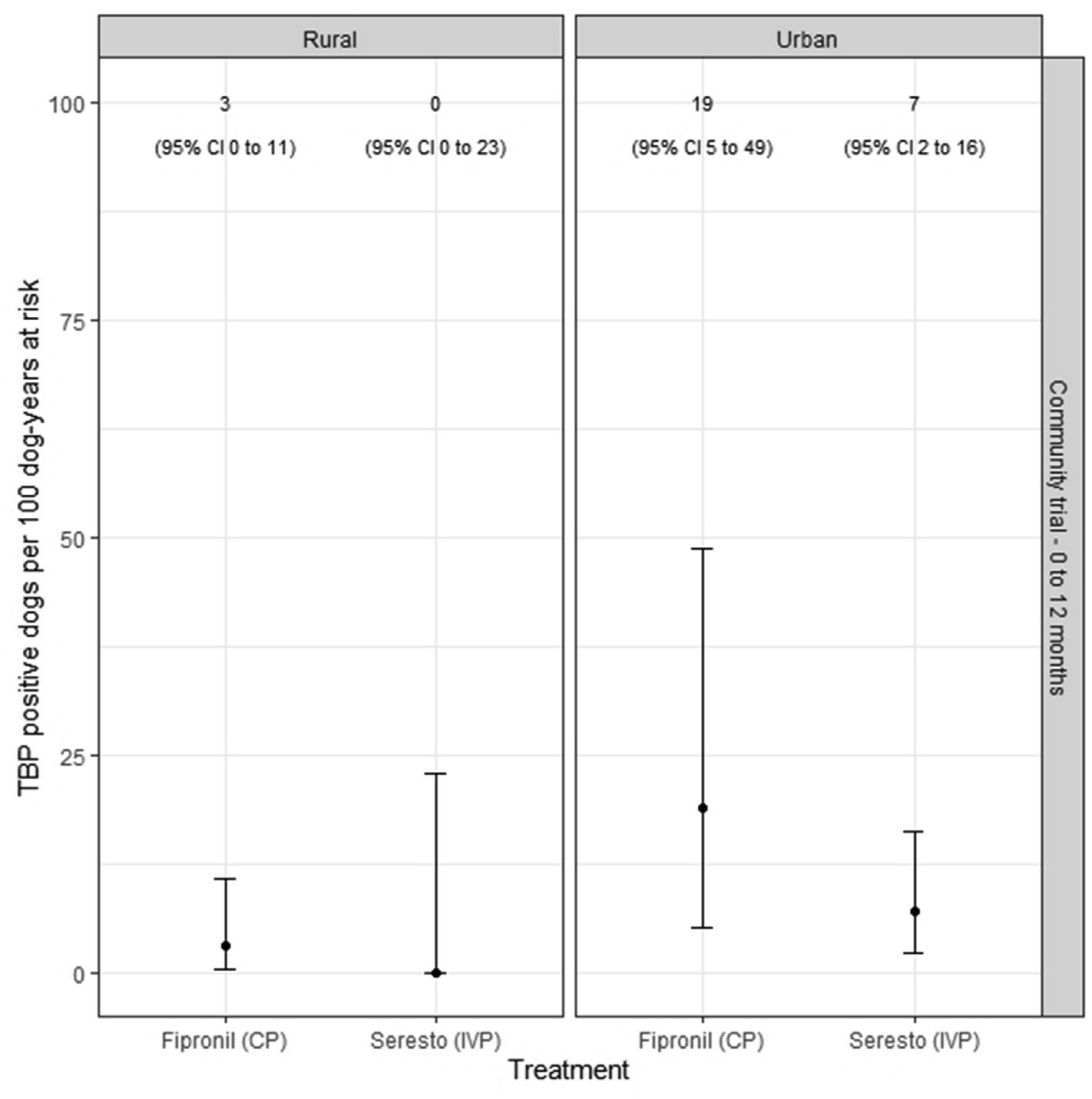
Error barplot showing the crude (unadjusted) tick-borne pathogen (TBP) incidence rate estimates, as assessed by qPCR, for dogs treated with CP (fipronil) and IVP (Seresto® collars), conditioned by environment (urban, rural). TBP incidence rate is expressed as the number of TBP positive dogs per 100 dog-years at risk. The error bars show the lower and upper bounds of the 95% confidence interval (CI) for the TBP incidence rate estimates.

Figure 4 shows the crude TBP incidence rate estimates for IVP- and CP-treated dogs, conditioned by the environment in which the dog was kept (urban, rural). After adjusting for the effect of environment there were 11 (95% CI: 4–26) TBP-positive CP dogs per 100 dog-years at risk compared with 3 (95% CI: 1–15) TBP-positive IVP dogs per 100 dog-years at risk. CP-treated dogs were 2.9 (95% CI: 0.8–10.4) times more likely to be TBP-positive compared with IVP-treated dogs. We estimate that 13 (95% CI: 9–40) dogs needed to be treated with the IVP (Seresto®) to avoid one case of TBP.

## 4 Discussion

### 4.1 Baseline assessment comparing topical and systemic ectoparasiticides

The collection of canine blood samples and relevant metadata at enrolment for this study provided us with the data necessary to complete a baselines assessment of apparent TBP prevalence in dogs using either a topical or systemic ectoparasiticide prior to the study’s commencement. At enrolment, 118 dogs (63%) were on a topical ectoparasiticide, which acts from the dog’s dermis and hair to prevent ectoparasite feeding, whilst 61 dogs (33%) were on a systemic ectoparasiticide which kills ectoparasites only after they have begun blood-feeding and seven dogs (4%) were on no ectoparasiticide.

We used three separate analyses to compare the effect of topical, systemic and no ectoparasiticide product used with TBP positivity as determined by qPCR, whilst also adjusting for the potential impact of canine environment. An error barplot (Fig. 2) showed that pathogen positivity was higher in dogs using systemic products if they were from an urban environment where TBP infection pressure may be higher. Moreover, dogs on no ectoparasiticide had higher rates of TBP positivity than those on both systemic and topical treatments, demonstrating that any ectoparasiticide is better than none. Nonetheless, substantial overlap in 95% CI between product types and no product meant that differences in TBP positivity could not be demonstrated as statistically significant at the *P* < 0.05 level. Such results were supported by our calculations of direct adjustment for apparent TBP prevalence between groups that also factored in the impact of canine environment. These identified TBP positivity at 5 dogs per 100 at risk using systemic products compared to 2 dogs per 100 at risk for topically treated dogs and 14 dogs per 100 at risk for those on no ectoparasiticide. Overall, systemically treated dogs were 2.7 (95% CI: 0.5–15.0) times more likely to be TBP-positive compared to topically treated ones. Such results demonstrate the TBP transmission blocking impact of topical products that do not permit ectoparasite blood-feeding. Additionally, the NNTB calculations ascertained that after accounting for the effect of environment, 31 dogs would need to be treated with a topical product to avoid one case of TBP contraction, further emphasising the benefits of ectoparasiticides that act from the canine dermis and hair.

The comparison of TBP-exposed dogs between those in the topical, systemic and no ectoparasiticide groups, as determined by serology, mirrored the results gleaned through qPCR analysis of current infections. Dogs on systemic and no ectoparasiticide products in urban environments had higher levels of TBP-exposure than those on topical products, although this same pattern was not reflected in the dogs tested in rural environments (Fig. 3). However, when direct adjusted values were calculated that factor in the potentially confounding impact of dog environment, the same trend in product performance was identified as previously, showing that dogs on systemic products were more likely to be exposed (23 per 100 dogs at risk) than those on topical products (15 per 100 dogs at risk). Dogs on no ectoparasiticide product also had higher levels of TBP exposure (21 per 100 dogs at risk) than those on topical products.

Despite these results, the small sample size of dogs for which ectoparasiticide product data were available, in conjunction with the low numbers of TBP-positive and TBP-exposed dogs, meant that 95% CI for the direct adjustment values were relatively high with substantial overlap, making unequivocal conclusions hard to draw. A larger study, specifically exploring and comparing prevention of TBP transmission *via* the performance of topical *vs* systemic products, alongside those on no ectoparasiticide, would need to be conducted to more conclusively demonstrate a difference between these product types.

Even though our limited data may only be indicative of a difference in TBP blocking impact of topical, systemic and no ectoparasiticide products, these tentative conclusions are supported by existing research. Jongejan et al. (2016) compared the *E. canis* blocking impact of two systemic products (afoxolaner and fluralaner) and one topical product (a spot-on combination of permethrin and imidacloprid) by *R. sanguineus* (*s.l.*) ticks in a laboratory controlled comparative efficacy study. Whilst the topical product consistently demonstrated a higher speed of kill and anti-attachment effect on ticks, its most valuable impact was its 100% blocking efficacy on *E. canis* transmission, whilst the blocking efficacy for the systemic product afoxolaner was 33.3% and for fluralaner was 66.7%. The authors concluded that speed of kill of the systemic products tested was not rapid enough to prevent *E. canis* transmission, which begins in under 8 h of tick attachment but may be as rapid as just a couple of hours (Fourie et al., 2013c; Jongejan et al., 2016).

Additionally, a study by Varloud et al. (2018a) identified similar results when comparing the blocking efficacy of transmission of *B. canis* by *Dermacentor reticulatus* ticks between systemic and topical ectoparasiticides. In this study, the orally administered systemic product (fluralaner) offered no protection from *B. canis* transmission, whilst dogs on the topical product (a combination of dinotefuran, permethrin, pyriproxyfen) had a five times lower risk of contracting this infection Varloud et al. (2018a). Nonetheless, similar research investigating the *B. canis* transmission blocking efficacy of afoxolaner and fluralaner have found it to be 100% effective at preventing contraction of this TBP during challenge trials (Beugnet et al., 2014; Taenzler et al., 2015). Differences in results between these studies may be because in the Varloud et al. (2018a) investigation *D. reticulatus* ticks were allowed to feed on donor sheep and become engorged prior to infestation on dogs, whilst unengorged ticks were used in the latter two studies. Tick engorgement may speed up TBP transmission with pre-engorged *D. reticulatus* having been demonstrated to transmit *B. canis* within 8 h of feeding commencement (Varloud et al., 2018b). Therefore, despite multiple factors impacting canine TBP transmission times, an ectoparasiticide approach that prevents ectoparasite feeding may always be optimal, as it limits any risk of TBP transmission, unlike systemic products.

### 4.2 Prospective community trial comparing two topical ectoparasiticides

The data from the full 12 months of our prospective community trial comparing the TBP-blocking efficacy of Seresto® in the IVP group and fipronil in the CP group found both products to be highly effective at preventing the contraction of canine TBP. Of the 164 dogs from which data was collectable at the study’s end, we found that just 2% in the CP group and 1% in the IVP group acquired a new TBP within the first 6 months, whilst 4.8% in the CP group and 3.7% in the IVP group contracted a new TBP between 6 and 12 months (Table 4). Given the high efficacy of fipronil in the CP group, the relative TBP blocking efficacy of Seresto® was just 49% as defined by Marchiondo et al. (2013) in the first 6 months of the trial and 23% in the second 6 months. Prior research supports data found in the present study, that fipronil is a highly effective product at preventing ectoparasite infestation, in species such as *R. sanguineus* (*s.l.*), *D. reticulatus*, *Ctenocephalides felis* and *Trichodectes canis* as well as blocking the pathogens they transmit, for example *E. canis* in field settings (Davoust et al., 2003; Kužner et al., 2013). Fipronil has also been demonstrated to cause salivary gland degeneration and prevention of engorgement in some tick species, thereby providing an additional mechanism by which TBP transmission is blocked (Pereira et al., 2009). Regarding prevention of ectoparasite infestation, these two products performed equally well, with no dogs in either group being found with ticks attached nor engorged at either the 6-month or 12-month time-points.

After adjusting for the effect of dog environment, we found that the rate of TBP-positivity was higher in the CP group at 11 (95% CI: 4–26) positive dogs per 100 dog-years at risk in comparison to 3 (95% CI: 1–15) positive dogs per 100 dog-years at risk in the IVP group, indicating a benefit conferred by the Seresto® collars over the fipronil used in the CP group. Expressed in another way, the incidence rate of TBP positivity for the CP group was 2.9 (95% CI: 0.8–10.4) times that of the IVP group (Seresto®). For both these findings, the error barplot showing unadjusted TBP incidence (Fig. 4) may highlight that this difference in performance is being driven by a superior TBP blocking efficacy by the IVP group (Seresto®) in the urban environment, whilst TBP incidence is similar for both groups in the rural environment. However, for both the crude and adjusted incidence rates the substantially overlapping 95% CI indicate that there is potentially only a marginal benefit conferred by Seresto® in TBP-blocking performance when directly compared to fipronil. A greater investigational cohort size and a longer sampling period over multiple cycles of Seresto® collar functional duration may be required to identify if such apparent differences in TBP-blocking performance can be further supported. Despite this, both ectoparasiticides performed well across the course of this trial and therefore either Seresto® or fipronil represent an efficacious product for protecting canine TBP contraction in the tropics.

Additionally, the number needed to treat for benefit (NNTB) identified through this community trial was 13, meaning 13 dogs would need to be treated with the IVP (Seresto®) to see one dog benefit and not contract a TBP relative to the CP group. Given the modest price difference of $3 × 8 months = $24 USD for Detick *vs* $30 USD for an 8-month duration Seresto® collar, the NNTB value calculated may mean the small benefit in reduced TBP incidence that Seresto® confers is worth the slightly higher ∼$6 USD price, depending on the economic situation of the purchaser.

Further key information obtained through our community trial data was evidence of successful treatment of dogs that were *A. platys*- and/or *E. canis*-positive at baseline. Three dogs were found qPCR-positive to *A. platys* at baseline and another was found co-infected with *A. platys* and *E. canis*, all four of these were treated with doxycycline at 10 mg/kg SID for the relevant course duration and were found negative for these pathogens at 6 and 12 months. Prior studies have found doxycycline to be highly efficacious at treating canine *A. platys* infections, whilst the data for *E. canis* treatment is more equivocal (Gaunt et al., 2010; Mylonakis et al., 2019). There is some evidence of doxycycline failing to achieve complete cure, with molecular evidence of maintained *E. canis* DNA presence after a 4-week period of doxycycline treatment in some dogs (Mylonakis et al., 2019).

Missing data generated by dog dropout had the potential to impact on our statistical analyses particularly in the context of this study where the total number of dogs enrolled was relatively low (Shih, 2002; McNeish, 2017). Simplistic removal of missing data from the overall dataset poses a risk of biasing analyses and generating spurious conclusions regarding the benefit or not of a particular product (Shih, 2002; McCoy, 2017). Therefore, we followed the recommendations of McNeish (2017) and used multiple imputation to impute the likely TBP positivity status of dogs that dropped out of the prospective community trial before their 6- or 12-month follow-up. This was deemed a more suitable method for handling missing data values than using best- and worst-case analysis reporting as recommended by Jakobsen et al. (2017) due to the unequal numbers of dropouts between the rural and urban environments within both the IVP and CP groups. The disparity between dropouts within these covariates meant that worst-case scenario models inflated the TBP incidence rates for particular groups such as CP dogs in the rural environment and therefore biased the overall results when running a worst-case scenario model. In contrast, multiple imputation did not generate inflated numbers of TBP infections within the dogs that dropped out of the study, providing a more balanced estimate of the effect of treatment.

This study has some limitations which may have impacted the community study analysis. The most significant of these is that numerous canines in the IVP group ripped off or tore their Seresto® collars at some stage in the trial. Within the IVP group five dogs (6%) had broken their collars by the end of the trial, with average collar replacement times taking 1–2 days. Two of the IVP group dogs found to have broken their collars at some point within the trial were found positive for a TBP: one to *A. platys* at 6 months and one to *B. vogeli* at 12 months. Therefore, whether such breakages may have had an impact on the effectiveness of the Seresto® collars, possibly by reducing the amount of active chemical on the dog’s skin for a few days and thereby reducing the collars ectoparasite repellency is difficult to discern. This also highlights the advantage of the topical fipronil used in the CP group, which does not have the issue of potential removal by the dog, although reapplication must be much more frequent than for the Seresto® collars at 1 *vs* 8 months.

Additional factors that may have impacted the efficacy of the Seresto® collars in the IVP group was the local Cambodian climate, that features a distinct wet monsoon season from May to September (Bairagi et al., 2020). Under conditions of regular rain exposure Seresto® collars have been demonstrated to show a significantly reduced efficacy at preventing tick and flea infestation, whilst topical fipronil does not show such a waning of efficacy under the same conditions (Halos et al., 2014; Beugnet et al., 2018). Taking this into consideration, the tropical field conditions our trial subjected the Seresto® collars to may have meant they were not working optimally and therefore reduced their TBP-blocking efficacy when compared to topical fipronil. Nevertheless, such data are critically important as the temperate climatic conditions Seresto® has been substantially tested in before may provide performance results that do not directly translate to tropical environment contexts (Stanneck et al., 2012c; Brianti et al., 2013; Dantas-Torres et al., 2013).

## 5 Conclusions

We have demonstrated that topical ectoparasiticides demonstrate an advantage over systemic isoxazoline products at reducing the contraction of canine TBP in Cambodia, within both rural and urban contexts. Such findings are critical to inform local veterinarians in the region that may commonly use systemic products, believing them to be effective at preventing TBP transmission to their clients’ dogs. Our data will be directly translated and disseminated to local veterinary practitioners, hopefully with the outcome of changing ectoparasiticide practices and reducing the amount of TBP circulation within this country. These findings are non-trivial, given the substantial pathogenicity of many of the TBP that systemic products are unable to block, particularly *E. canis* that frequently generates fatal canine disease (Little, 2010; Mylonakis et al., 2019). Secondly, our prospective community trial showed some benefit conferred by Seresto® collars over monthly topical fipronil at blocking TBP transmission, although both products were still highly effective. Final choice of ectoparasiticide product for canine protection in the region will need to be evaluated on a case-by-case basis as Seresto® may offer slightly enhanced protection, whilst Detick (fipronil) offers greater affordability and availability. Overall, both topical products should be chosen over systemic ectoparasiticides, which offer limited protection against TBP contraction for dogs in the tropics, thereby reducing the TBP burden of disease in pet and working mine detection dogs in Cambodia and across Southeast Asia.

## Funding

This study was funded by an Australian Research Council Linkage grant LP170100187 with Elanco GmbH as industry partners. Financial support was also provided by the University of Melbourne postgraduate scholarship scheme. The funders had no role in study design, data collection and analysis, decision to publish, or preparation of the manuscript.

## Ethical approval

This study was approved by the Office of Research Integrity and Ethics, University of Melbourne, Australia, under ethics permit 1814620.

## CRediT author statement

Lucas G. Huggins: data curation, sample processing, project administration, formal analysis and validation, writing – original draft, reviewing & editing. Mark Stevenson: formal and statistical analyses, writing – reviewing & editing. Zahida Baydoun: sample collection and project administration, writing – reviewing. Ron Mab: sample collection and writing – reviewing. Yulia Khouri: project administration and resources, writing – reviewing. Bettina Schunack: project administration, writing – reviewing and funding. Rebecca J. Traub: project conceptualization, funding, administration, resources, writing – review & editing. All authors read and approved the final manuscript.

## Data availability

All datasheets, metadata, qPCR and serological results are available from the authors upon request.

## Declaration of competing interests

Lucas G. Huggins, Mark Stevenson, Zahida Baydoun, Ron Mab, Yulia Khouri and Rebecca J. Traub have conducted research funded by Elanco GmbH. Bettina Schunack is an employee of Elanco GmbH.
